# CHRONIC PHYSICAL HEALTH CONDITIONS, DEPRESSIVE SYMPTOMS, AND SELF-RATED HEALTH IN GRANDMOTHERS

**DOI:** 10.1093/geroni/igac059.713

**Published:** 2022-12-20

**Authors:** Christina Henrich, Carol Musil, Jaclene Zausniewski, Christopher Burant, Elizabeth Tracy, Alexandra Jeanblanc

**Affiliations:** Case Western Reserve University, Cleveland, Ohio, United States; Case Western Reserve University, Cleveland, Ohio, United States; Case Western Reserve University, Cleveland, Ohio, United States; Case Western Reserve University, Parma, Ohio, United States; Case Western Reserve University, Cleveland, Ohio, United States; Case Western Reserve University, Cleveland, Ohio, United States

## Abstract

Grandmothers often participate in caregiving to grandchildren, ranging from informal caregiving to having responsibility for raising grandchildren, but health problems may affect their ability to do so. To better understand potential health problems in grandmother caregivers, this secondary analysis examined 1) longitudinal relationships between chronic physical health conditions, depressive symptoms, and self-rated health with race, age, & marital status, and 2) causal ordering of depressive symptoms and self-rated health, controlling for baseline race, age, marital status, and chronic conditions of 328 Ohio grandmothers across 3 time points over 2 years. The study was guided using Lenz’s Theory of Unpleasant Symptoms. Measures included chronic physical health conditions (modified Charlson), depressive symptoms (CES-D), self-rated health (SF-36), and demographic variables of race (white/nonwhite), age, and marital status (married-partnered/not married). Analyses included correlations and structural equation modeling. Pearson product moment correlations indicated significant relationships (ranging from 0.26 to 0.56) between chronic conditions, depressive symptoms and self-rated health at all three time points. Next, a bivariate autoregressive model was tested using structural equation modeling. The data fit the model well. The goodness of fit results included: Chi square=55.39, TLI=0.94, CFI=0.97, and RMSEA=0.07. Results indicated consistency of depressive symptoms and self-rated health over time, and these were affected by chronic conditions; the causal order indicated that self-rated health impacts depressive symptoms. This study contributes new knowledge about the relationships and causal sequence of self-rated health and depressive symptoms. Implications on practice and research are discussed.

